# The Effect of Volatile Oil from* Vernonia anthelmintica* Seeds on Melanin Synthesis in B16 Cells and Its Chemical Analysis by GC-QTOF-MS

**DOI:** 10.1155/2018/6291281

**Published:** 2018-08-07

**Authors:** Abulikemu Aobuli, Jumai Maitusong, Mahinur Bakri, Xueying Lu, Maitinuer Maiwulanjiang, Haji Akber Aisa

**Affiliations:** ^1^The Key Laboratory of Plant Resources and Chemistry of Arid Zone, Xinjiang Technical Institute of Physics and Chemistry, Chinese Academy of Sciences, Urumqi 830011, China; ^2^University of The Chinese Academy of Sciences, Beijing 100039, China; ^3^State Key Laboratory Basis of Xinjiang Indigenous Medicinal Plants Resource Utilization, Xinjiang Technical Institute of Physics and Chemistry, Chinese Academy of Sciences, Urumqi 830011, China

## Abstract

*Vernonia anthelmintica* Willd. seeds have been used in folk medicine for the treatment of leukoderma in Xinjiang, China, for more than 300 years. The promoting activities of its volatile oil (AVO) in melanogenesis and its chemical composition were investigated in this paper. The bioactivities of AVO were examined by melanin synthesis and tyrosinase activity assay in B16 cells. Using GC-QTOF-MS technology, each compound of AVO contains a single separated peak in GC and the retention indices of every GC peak were calculated by the retention times of C7~C30* n*-alkanes that were injected at the same chromatographic conditions. Then each individual peak was identified by comparing its mass spectrum with the MS library (NIST 14). As a result, AVO increased the melanin content and tyrosinase activity in a dose-dependent manner at concentrations of 10-30*μ*g·mL^−1^. The 64 compounds were identified in AVO which occupied 95.15% of total peak area in GC. They mainly contained caryophyllene (23.73%), sabinene (18.15%), *α*-thujene (6.57%), thymol (5.29%), 4-epi-*α*-acoradiene (4.98%), limonene (4.92%), anethole (3.44%), etc. According to the results the AVO can promote melanogenesis and upregulate tyrosinase activity in B16 cells.

## 1. Introduction

*Vernonia anthelmintica* Willd., a member of the family Asteraceae, is an erect forb or shrub where more than 1000 species of the genus* Vernonia* were widely distributed in subtropical and tropical areas throughout Asia and Africa [1–3]. In traditional Uyghur medicine (TUM), seeds of the* Vernonia anthelmintica* Willd. (*V. anthelmintica*) were used as a herbal medicine with the common name of “Kaliziri” for the treatment of diabetes mellitus, leukoderma, skin disease, fever, worm infection, and kidney trouble [4].

In recent years with the development of traditional medicine in China,* V. anthelmintica *has been widely used in the medicinal formula for the treatment of vitiligo, such as “Kursi babuqi” and “Injection of Kaliziri.” Wang et al. [5] treated 157 patients with “Kursi babuqi” for three months and that showed that effective rate of treatment group was 56%. Liu et al. [6] reported that 445 patients of vitiligo vulgaris were treated with “Kursi babuqi” for one year, and effectiveness of treatment reached 93.5%. For the other formulation of* V. anthelmintica*, “injection of Kaliziri” was applied to the patient combined with UV treatment, and effective rate of treatment group was 70.4% [7]. The herb also was recorded in the book of “Ministry of PRC Health and Drug standards” [8, 9] and also was recorded with various biological effects, including antiarthritic [1], antibacterial, anthelmintic, analgesic, anti-inflammatory, antioxidant, antidiabetic, antihyperlipidemic, and cytotoxic activities [10–15].

Previous phytochemical investigations of the herb have shown that it contains flavones [16], caffeoylquinic acid derivatives [17], sesquiterpene, triterpenoids, elemanolide sesquiterpenoids, and guaianolide sesquiterpenoids [1]. It was reported that few compounds were isolated from the herb which able to promote melanin synthesis activity [18, 19].

Although many researches about* V. anthelmintica *seeds were found, no more scientific results were reported about the chemical composition and bioactivity of volatile oil from* V. anthelmintica* seeds (AVO). To search active components for the treatment of vitiligo we studied volatile components of* V. anthelmintica* and its bioactivities. In this research, we studied the AVO effects on melanogenesis in B16 cells and investigated its chemical composition by GC-QTOF-MS method.

## 2. Materials and Methods

### 2.1. Plant Material

The seeds of* V. anthelmintica* (batch number: Z30031501) were purchased in March 2016, from Xinjiang Meditsina Uyghur pharmaceutical Co., Ltd, Xinjiang, China. Voucher specimen has been deposited at the herbarium (Voucher no. WY02316), Xinjiang Institute of Ecology and Geography. Plant materials were identified by Professor Feng Ying from Xinjiang Institute of Ecology and Geography, Chinese Academy of Science.

### 2.2. Reagents

DMSO (D8418) was purchased from Sigma-Aldrich (St. Louis, MO, USA). A cell counting kit (FC101) was purchased from TransGen Biotechnology (Beijing, China). C7-C30 saturated alkanes standard 1ml (SUPELCO, USA),* n*-hexane (analytical grade).

### 2.3. Extraction and Sample Preparation

The powdered* V. anthelmintica* seeds (100g) were extracted by volatile oil extractor with 1400 mL water for 6h. The oil was collected and dried over anhydrous sodium sulfate and dissolved in dimethyl sulfoxide (DMSO) to make a stock solution and then stored at -20°C until use. 50mg·mL^−1^ stock solution of AVO in DMSO was used for cell experiment. Fourfold dilution of AVO with* n*-hexane was used for chemical analysis.

### 2.4. Cell Culture

The murine B16 melanoma cells were purchased from the Type Culture Collection of the Chinese Academy of Sciences (Shanghai, China, No. TCM2) and were cultured in high glucose Dulbecco's modified eagle medium (HG-DMEM, Gibco) supplemented with 10% FBS (BI, Biological Industries), 100 *μ*g•mL^−1^ streptomycin, and 100 U•mL^−1^ penicillin (Gibco, Cat. No. 15140-122). The cells were maintained in a humidified incubator with 5% CO2 at 37°C, and they were subcultured every 2 days to maintain logarithmic growth.

### 2.5. Cell Viability Assay

The viability of cultured B16 cells was assayed by adding CCK-8 solution. Generally, B16 cells were plated in 96-well plate at a density of 5×10^3^ cells per well and incubated for 24 h. Test samples were added with different concentration, and the cells were incubated for 24h. After discarding the culture medium of the cells, 10*μ*L of CCK-8 solution was added into each well and the cells were incubated at 37°C for 2h. The absorbance was determined at 450 nm using microplate spectrophotometer (Spectra Max M5, Molecular Devices Company, Sunnyvale, CA, USA). The absorbance of cells without treatment was regarded as 100% of cell survival. Each treatment was performed in triplicate, and each experiment was repeated for three times.

### 2.6. Melanin Content Assay

The B16 cells were seeded in a six-well plate (2×10^5^ cells/ well) for 12h, after renewing the culture medium of the cells treated with different concentrations of AVO for 48h and washed with ice-cold PBS twice. Cells were lysed [16], and each lysate was taken 150*μ*l into a 96-well plate and measured; optical density of the solubilized melanin was measured at 405 nm by a multiplate reader. BCA Protein Assay Kit (Biomed, Beijing, China) was used to determine the protein concentration of each sample. The melanin content was normalized to the cellular protein concentration (abs melanin/*μ*g protein). The percentage value of the AVO treated cells was calculated with respect to the negative control [20].

### 2.7. Tyrosinase Activity Assay

The tyrosinase activity was estimated by measuring the rate of L-DOPA oxidation [21, 22]. B16 cells were seeded in a six-well plate at a density of 3×10^5^ cells per well and allowed to attach for 12h. Cultured B16 cells were incubated in the absence or presence of AVO for 24h, and the adherent cells were washed with ice-cold PBS twice and lysed with 100*μ*L lysis buffer (1% sodium deoxycholate and 1% TritonX-100 in PBS) for 30 min at 80°C, and each lysate was centrifuged at 12,000×g for 15 min to obtain the supernatant. Ninety microliters (90 *μ*L) of the supernatant was added to 10 *μ*L of freshly prepared 10 mmol·mL^−1^ L-DOPA solution in each well of a 96-well plate. Then, the cells were incubated at 37°C in dark for 30 min and the absorbance of dopachrome was measured at 490 nm using a microplate reader. Data was calculated using the following formula: tyrosinase activity (%) = (OD 490 of sample/OD 490 of control) × 100.

### 2.8. GC-QTOF-MS Analysis

For the chemical analysis of AVO, an Agilent 7200 GC-QTOF-MS (Agilent, SantaClara, USA) was used, equipped with a HP-INNOWax capillary column (Agilent, 60 m×0.25 mm i.d., 0.25 *μ*m film thickness, polyethylene glycol stationary phase). The carrier gas was 99.99% high purity Helium with a flow rate of 0.8 mL·min^−1^. GC injection was performed in pulsed split mode (split rate 10:1, purge flow to split vent 5ML·min^−1^). The following GC temperature gradient was used: 60°C for 2 min, 2°C/min to 80°C, 8°C/min to 180°C, 4°C/min to 240°C, and holding at 240°C for 30min. The temperatures of the injector, the transfer line, and the ion source were set to 250, 280, and 230°C, respectively. The total run time was 65 min with a solvent delay of 5 min. The QTOF mass spectrum was recorded at five scans per second with a mass-to-charge ratio 50-500 m/z mass acquisition range. Ionization was operated in electron impact ionization (EI) mode at 70 eV. 1.2*μ*L AVO sample solution was injected in to GC-QTOF-MS system for analysis, in the same chromatographic conditions that C7-C30* n*-alkanes standard used for calculating the retention times indices of GC peak from sample (Supplementary Figure 2) [23, 24].

### 2.9. Data Analysis

The results of cell experiment were expressed as the mean ± SD, and the statistical analysis was performed by one-way ANOVA followed by a Tukey post hoc test for multiple comparison tests. Significant differences were accepted when P < 0.05. The retention indices (I) of every GC peaks were calculated with retention times of C7-C30* n*-alkanes standard that were injected at the same chromatographic conditions. Then, the mass spectral data were processed using Mass Hunter Qualitative Analysis B.070. The compounds were identified by comparing with mass spectra NIST14. L library data [23] and validated with the published compounds index data. The relative percentage of compounds was normalized by peak area.

(1) $$\begin{array}{c} I=100\times \frac{lg\;t^{/}\left(i\right)-lg\;t^{/}\left(n\right)}{lg\;t^{/}\left(n+1\right)-lg\;t^{/}\left(n\right)} \end{array}$$

where *I* is the retention index of the components to be tested and* (i)* is the adjusted retention time (min) of the components to be tested;* n* and* n+*1 are the carbon amounts of* n*-alkanes before and after the unknowns effused, respectively; *t*′*(n)* and *t*′*(n+*1) are the No.* n* and No.* n+*1 carbon retention times of* n*-alkanes, respectively [25, 26].

## 3. Results

### 3.1. Cell Viability of Melanoma Cells Induced by AVO

**Figure 1:**
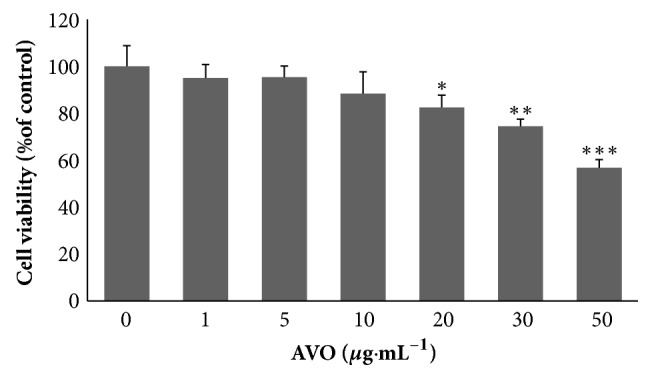
B16 melanoma cells were exposed to various concentrations of AVO (1, 5, 10, 20, 30, and 50*μ*g·mL^−1^) for 24h. Cell viability was measured by a CCK-8 assay. The data are shown as the means ± SD;* n*=3.

Our results showed that murine melanoma B16 cells treated with concentration of 1-10*μ*g·mL^−1^ AVO for 24h did not induce any changes in cell viability. At the concentration of 10-30*μ*g·mL^−1^, the cell viability was around 80 to 90 percent which display slight cytotoxicity. Compared with untreated cells, with the treatment of 50*μ*g·mL^−1^ of AVO cell viability was only 56 percent and indicated relative higher cytotoxicity (Figure 1). Therefore, the concentrations at 10-30*μ*g·mL^−1^ are suitable for further evaluating the effects of AVO on tyrosinase activity and melanin synthesis in B16 cells.

### 3.2. AVO Enhances Intracellular Melanin Content and Tyrosinase Activity

**Figure 2:**
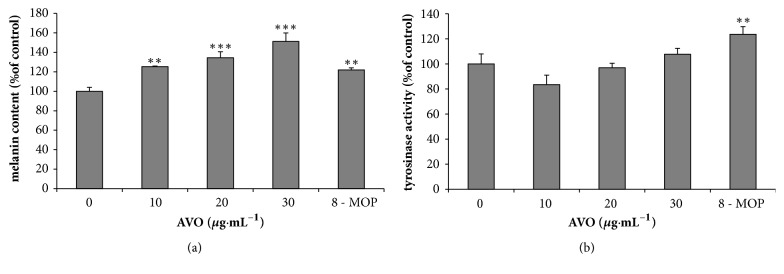
Cellular melanin synthesis (a) in B16 melanoma cells and concentration-dependent effect of AVO on tyrosinase activity (b). Cells were treated with 0.1% DMSO as a vehicle or with AVO at 10, 20, and 30*μ*g·mL^−1^ and 50*μ*M 8-MOP as a positive control. The data of melanin content is shown (a). The cells were then analyzed by tyrosinase activity assay (b). Each percentage value for treated cells is reported relative to that of 0.1% DMSO cells. The results have been shown as the means ± SD; n = 3, *∗∗* p <0.01, and *∗∗∗* p <0.001 compared with 0.1% DMSO cells.

In the melanin content assay, the absorbance of the same number of cells across AVO concentrations (10-30*μ*g·mL^−1^) was measured in order to exclude the possibility that a rise in melanin content may be induced by the cell proliferating effect of AVO. We found that melanin levels were increased in B16 cells in a dose-dependent manner after the treatment of AVO (Figure 2(a)). At the concentration of 10-30*μ*g·mL^−1^, the melanin content showed concentration-dependent increase, while the concentration of 20-30*μ*g·mL^−1^ showed significant increase in melanin content. The effect of AVO on tyrosinase activity did not show significant difference compared with untreated group (Figure 2(b)); however it showed a rise trend.

### 3.3. GC-QTOF-MS Analysis

**Figure 3:**
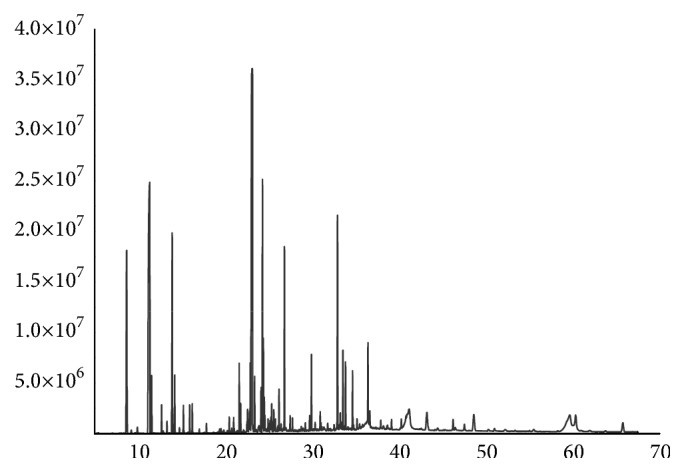
The GC peak spectra of AVO.

The result of AVO was obtained at a yield of 0.15% w/w. The 86 peaks were separated in GC as shown Figure 3 and preliminarily identified 64 compounds by QTOF-MS which account for 95.15% of total peak area (Table 1). According to the chemical composition analysis of AVO, the terpenoids accounted for 73.53%, phenolics 8.07%, ethers 5.31%, esters 3.76%, hydrocarbons 3.54%, aromatics 0.42%, ketones 0.39%, and acids 0.15%.The main chemical components of AVO were caryophyllene 23.73%, sabinen18.15%, *α*-thujene 6.57%, thymol 5.29%, limonene4.92%, and 4-epi-*α*-acoradiene 4.98%.

## 4. Discussion

Vitiligo is an acquired, progressive, multifactorial, and depigmentation disorder characterized by the appearance of circumscribed white macules in the skin caused by chronic, progressive loss of functional melanocytes in the epidermis [27, 28]. The etiology of vitiligo is poorly understood. Also, the numerous factors have been implicated in the development of vitiligo, including trauma, stress, exposure to sunlight for long time, skin infections, neural stress, cancer, melatonin receptors dysfunction, some drugs, and endocrine diseases and toxic compounds. These causal factors may act independently or in concert [29]. The pigmentation of the skin provides many valuable function; perhaps foremost among these is the photo protection of underlying tissues from ultraviolet (UV) radiation. Melanocytes respond to a wide variety of intrinsic and extrinsic factors produced by the environment or by neighboring cells in the skin, including UV, stimulating hormone (MSH), agouti signal protein (ASP), endothelin 1 (ET1), dickkopf 1 (DKK1), a wide variety of growth factors, and cytokines [4]. Melanin biosynthesis is catalyzed by three melanocyte-specific enzymes: TYR, tyrosinase-related protein 1 (TRP-1), and TRP-2 [4]. The tyrosinase playing an essential role in melanocyte melanin biosynthesis has been known for many decades. Therefore, tyrosinase is an important index for the treatment and diagnosis of vitiligo. In our result the AVO increased the melanin synthesis and slightly impacted tyrosinase activity but did not make a significant difference with untreated group, so, we considered that AVO can promote melanin biosynthesis may relate with other enzyme (TRP-1, TRP-2). It provided potential research value.

Melanocyte loss and its underlying pathogenic mechanisms are not fully understood [30]. Both intrinsic (gene polymorphisms/mutations, autoimmunity, autotoxicity of melanocytes, and oxidative stress) and extrinsic factors are known to contribute to vitiligo, and it is recognized that hypersensitivity to oxidative stress has an important role in ultimate destruction of epidermal melanocytes. Oxidative stress has been demonstrated to be enhanced in pathological melanocytes and closely related to melanocyte apoptosis and autoimmune destruction [31–36]. Our preliminary result also suggests that AVO could protect B16 cells from H_2_O_2_-induced oxidative damage (Supplementary Figure 1).

**Figure 4:**
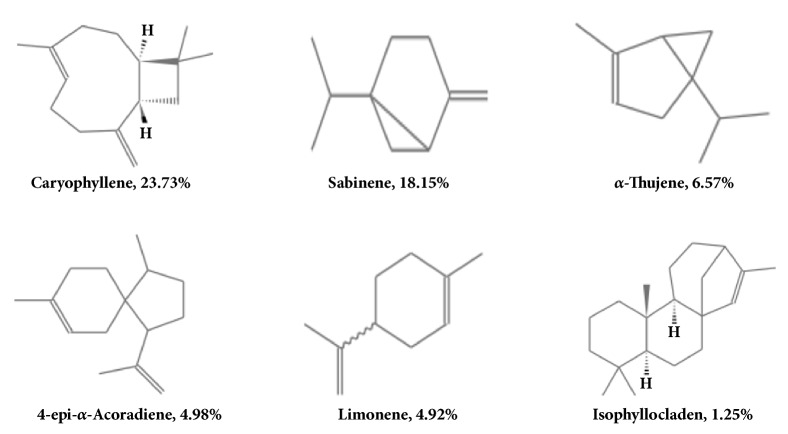
The structure of mainly terpenoids in AVO.

As volatiles, AVO was characterized by a strong odor. It is synthesized by plants as secondary metabolites and has been widely used owing to its bactericidal, virucidal, fungicidal, antiparasitic, insecticidal, anticancer, antioxidant, antidiabetic and cardiovascular effects [37]. The AVO was mainly composed of terpenoids occupying 73.53% of total chemical content, such as caryophyllene, sabinene, and limonene. Being natural compounds, the terpenoids were shown to have wide range of biological and pharmacological activities, including central effects, antimicrobial, and antitumor actions [38, 39]. In our results, high concentrations of AVO showed little cytotoxic properties in melanocytes. Therefore, we observed the protective effect of AVO on H_2_O_2_-induced B16 cells injury. Result showed that pretreatment of AVO reduced H_2_O_2_-induced cell injury in a dose-dependent manner (supplementary Figure 1). From our result, we tentatively conclude that AVO could protect melanocytes from harmful factors in vitiligo like oxidative stress. This result is similar to the antitumor and antioxidant effect of terpenoids. So, we considered that the terpenoids were major biologically active compounds in AVO. Since AVO had possessed terpenoids such as caryophyllene, sabinen, 4-epi-*α*-acoradiene, *α*-thujene, and limonene, it is hypothesized that the unsaturated double bond in active ingredients of AVO may be responsible for its antioxidant properties (Figure 4). Caryophyllene as a main content in AVO provided extensive pharmacological effects. Dahham et al. [37] reported that compounds have anticancer, antioxidant, and antimicrobial activities. They observed the antiproliferative effect of caryophyllene on the cancer cell lines. Among the tested cancer cells, the compound demonstrated selective antiproliferative effect against three cancer cell lines, namely, HCT 116, PANC-1, and HT29 cells, whereas it exhibited either moderate or poor cytotoxic effects against ME-180, PC3, K562, and MCF-7. Noteworthily, the compound displayed low toxicity against the normal cell lines such as 3T3-L1 and RGC-5. The study concluded that caryophyllene has strong selective cytotoxic properties against human colorectal cancer cells. Based on this report and our result of AVO cell viability assay, we considered that caryophyllene may be responsible for AVO's biological effect. In addition, we employed the TCMSP database to check the ADME properties and targets for the main compounds of AVO (http://lsp.nwu.edu.cn/molecule.php?qn=193). Data showed that caryophyllene and limonene related some autoimmune diseases, genetic diseases, and corresponding targets (supplementary Table 1), such as cardiovascular disease, arthritis, cancer, autoimmune cardiomyopathy, and Parkinson's disease. From the database, we could not correlate the exact relation between AVO active components and vitiligo or melanin loss disease. But according to the database information, we inferred that caryophyllene and limonene has immunomodulatory, antioxidant, and anticancer effects. Hence, we hypothesized that AVO increased melanin biosynthesis may be related to immunomodulatory and antioxidant effect from caryophyllene and limonene.

## 5. Conclusions

According to the biological and chemical analysis of AVO, the AVO can increase melanin synthesis. It be may concluded that terpenoids rich chemical properties of AVO may be responsible for its biological effects.

## Figures and Tables

**Table 1:** Volatile compositions in AVO.

	Compound	RT(min)	Possible formula	RI	Content (%)
1	*α*-Thujene	8.68	C_10_H_16_	1029	6.57
2	Toluene	9.22	C_7_H_8_	1049	0.08
3	Camphene	9.92	C_10_H_16_	1075	0.17
4	Sabinene	11.34	C_10_H_16_	1124	18.15
5	*β*-Thujene	11.56	C_10_H_16_	1131	1.08
6	*α*-Phellandrene	12.71	C_10_H_16_	1169	0.53
7	*α*-Terpine	13.33	C_10_H_16_	1189	0.22
8	Limonene	13.92	C_10_H_16_	1209	4.92
9	Eucalyptol	14.21	C_10_H_18_O	1219	1.23
10	Methoxyacetic acid,4-tetradecyl ester	14.76	C_17_H_34_O_3_	1239	0.11
11	*γ*-Terpinene	15.22	C_10_H_16_	1255	0.48
12	o-Cymene	15.94	C_10_H_14_	1281	0.47
13	Terpinolene	16.24	C_10_H_16_	1292	0.47
14	Isomenthone	20.5	C_10_H_18_O	1481	0.28
15	Copaene	21.00	C_15_H_24_	1507	0.22
16	Linalyl acetate	21.64	C_12_H_20_O_2_	1543	0.94
17	*α*-Gurjunene	21.80	C_15_H_24_	1552	0.47
18	Fenchol	22.53	C_10_H_18_O	1593	0.17
19	*β*-Elemene	22.6	C_15_H_24_	1596	0.43
20	cis-Caryophyllene	22.83	C_15_H_24_	1610	0.45
21	L-terpinen-4-ol	22.92	C_10_H_18_O	1615	0.99
22	Caryophyllene	23.14	C_15_H_24_	1628	23.73
23	dl-Menthol	23.42	C_10_H_20_O	1645	0.83
24	Ginsinsene	24.16	C_15_H_24_	1689	0.86
25	4-epi-*α*-Acoradiene	24.32	C_15_H_24_	1699	4.98
26	*α*-Terpinyl acetate	24.42	C_12_H_20_O_2_	1705	1.38
27	Chamigren	24.52	C_15_H_24_	1711	0.51
28	*β*-Selinene	24.6	C_15_H_24_	1716	0.22
29	Valencene	24.96	C_15_H_24_	1738	0.20
30	Eremophilene	25.06	C_15_H_24_	1743	0.09
31	Bicyclogermacrene	25.18	C_15_H_24_	1750	0.18
32	*δ*-Selinene	25.22	C_15_H_24_	1753	0.11
33	Piperitone	25.3	C_10_H_16_O	1758	0.26
34	(-)-Carvone	25.37	C_10_H_14_O	1762	0.46
35	*δ*-Cadinene	25.62	C_15_H_24_	1777	0.33
36	Octadecane	25.81	C_18_H_38_	1788	0.20
37	Cuminal	26.22	C_10_H_12_O	1813	0.70
38	*α*-Terpinen-7-al	26.46	C_10_H_14_O	1827	0.14
39	Anethole	26.85	C_10_H_12_O	1849	3.44
40	2-Methyloctadecane	27.51	C_19_H_40_	1888	0.21
41	Isosafrole	27.77	C_10_H_10_O_2_	1903	0.21
42	Alloaromadendrene oxide-(2)	29.74	C_15_H_24_O	2015	0.27
43	Caryophyllene oxide	29.96	C_15_H_24_O	2026	1.39
44	Globulol	30.96	C_15_H_26_O	2081	0.35
45	Phenol, 2-methoxy-4-propyl-	31.83	C_10_H_14_O_2_	2128	0.16
46	Thunbergene	32.56	C_20_H_32_	2167	0.11
47	Thymol	32.95	C_10_H_14_O	2187	5.29
48	Verticiol	33.27	C20H34O	2205	0.30
49	Hexadecanoic acid, methyl ester	33.45	C_17_H_34_O_2_	2214	0.11
50	Carvacrol	33.58	C_10_H_14_O	2221	1.62
51	Isophyllocladen	33.88	C_20_H_32_	2236	1.25
52	Cembrene A	34.69	C_20_H_32_	2279	1.09
53	2,4-Di-tert-butylphenol	35.2	C_14_H_22_O	2305	0.17
54	*α*-Pinacene	36.46	C_20_H_32_	2371	1.57
55	Dill apiol	36.66	C_12_H_14_O_4_	2382	0.33
56	8,11,13-triene-18-Norabieta	37.94	C_19_H_28_	2446	0.16
57	3,3,4,5,5,8-Hexamethyl-3,5,6,7-tetrahydro-S-indacen-1(2H)-one	39.17	C_18_H_24_O	2507	0.20
58	*γ*-Thujaplicine	40.29	C_10_H_12_O_2_	2556	0.19
59	Methyl dehydroabietate	43.25	C_21_H_30_O_2_	2673	1.22
60	Kolavenol	46.26	C_20_H_34_O	2771	0.34
61	9-Ethyl-10-methylanthracene	46.50	C_17_H_16_	2778	0.10
62	10,18-Bisnorabieta-5,7,9(10),11,13-pentaene	47.56	C_17_H_16_	2809	0.19
63	Retene	48.65	C_18_H_18_	2836	1.12
64	n-Hexadecanoic acid	51.02	C_16_H_32_O_2_	2895	0.15

	**Total**				**95.15**

## Data Availability

Most of the data (Figures 1, 2, and 3 and Table 1) used to support the findings of this study are included within the article. Some of the data (Supplementary Figure : GC-MS chromatogram of n-alkanes) used to support the findings of this study are included within the supplementary information file.

## Supplementary Materials

Supplementary Materials Supplementary Figure 1: AVO prevents cell death in H_2_O_2_-treted B16 cells. Cell viability was measured by a CCK-8 assay. The data are shown as the means + SD; n=3 and *∗∗∗* p <0.001 compared with control group. Supplementary Figure 2: the GC peak of* n*-alkanes standard. Supplementary Table 1: related diseases and targets of caryophyllene and limonene.
