# Supplementary material for: The Effect of Volatile Oil from Vernonia anthelmintica Seeds on Melanin Synthesis in B16 Cells and Its Chemical Analysis by GC-QTOF-MS
Source: Evid Based Complement Alternat Med. 2018 Aug 7;2018:6291281. doi: 10.1155/2018/6291281 (PMC6106727; doi:10.1155/2018/6291281)
Supplement: Supplementary Materials — Supplementary Figure 1: AVO prevents cell death in H2O2-treted B16 cells. Cell viability was measured by a CCK-8 assay. The data are shown as the means + SD; n=3 and ∗∗∗ p <0.001 compared with control group. Supplementary Figure 2: the GC peak of n-alkanes standard. Supplementary Table 1: related diseases and targets of caryophyllene and limonene. [file 6291281.f1.pdf]

Supplementary Information

The Effect of Volatile Oil from *Vernonia anthelmintica* Seeds on Melanin Synthesis in B16 Cells and Its Chemical Analysis by GC-QTOF-MS

Abulikemu Aobuli[1, 3], Jumai Maitusong [2, 3], Mahinur Bakri [1, 2], Xueying Lu[1], Maitinuer Maiwulanjiang[1, 2*], Haji Akber Aisa[1*]

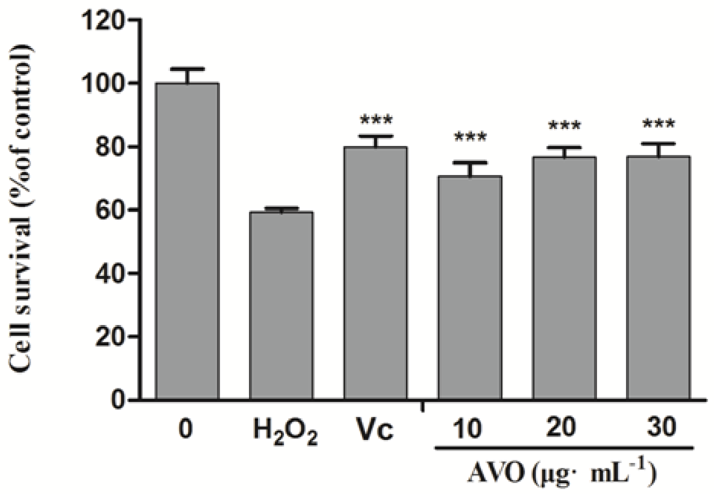

Supplementary figure 1 AVO prevent cell death in H_2O_2-treted B16 cells. Cell viability was measured by a CCK-8 assay. The data are shown as the means + SD; n=3, *** p <0.001 compared with control group.

Briefly, B16 cells were plated in 96-well plate and pre-treated with Various concentration(10, 20, 30 μg·mL-[1]) of AVO and Vc (1μM) for 24h. Then, the cultures were treated with H_2O_2 concentration of 250uM for 4h. The viability of cultured B16 cells was assayed by adding CCK-8 solution, used method 2.5.

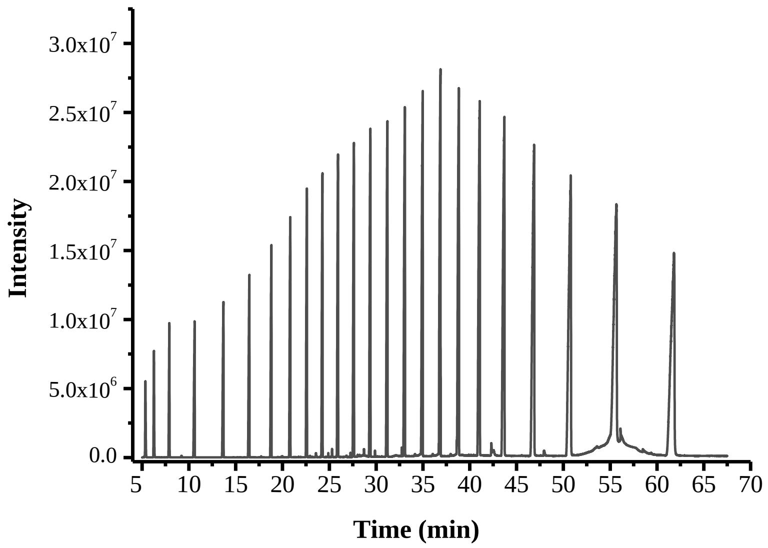

Supplementary figure 2 The GC peak of *n*-alkanes standard

The retention indices (I) of every GC peak from AVO were calculated with retention times of C7-C30 *n*-alkanes standard that were injected at the same chromatographic conditions. Then, the mass spectral data were processed using Mass Hunter Qualitative Analysis B.070. The compounds were identified by comparing with mass spectra NIST14. L library data and validated with the published compounds index data.

Supplementary table 1: Related diseases and Targets of Caryophyllene and Limonene

Name	Related disease	Target
Caryophyllene	Cardiovascular disease, Chronic inflammatory diseases, Airway hyperreactivity , Urge incontinence, Alzheimer's disease, Cognitive deficits, Schizophrenia, Abdominal aortic aneurysm , Analgesics , Arthritis, Bladder cancer , Breast cancer, Cancer, Unspecific carcinoma in situ, Colorectal cancer, Dysmenorrhea, Genitourinary tumors, Gestational hypertension, Inflammatory diseases, Lung Cancer, Malignant mesothelioma, Meningioma, Myocardial Infarction, Oropharyngeal squamous cell carcinoma, Osteoarthritis, Pain, unspecified, Pathological angiogenesis, Peutz-Jeghers syndrome, Prostate cancer, Pyresis, Renal Cell Carcinoma, mRheumatoid arthritis, Autoimmune cardiomyopathy , Chronic obstructive pulmonary disease, unspecified, Hypothermia, Neurogenic bladder, Tremor, unspecified ,Shy-Drager syndrome, Anesthesia, Anxiety Disorders, Insomnia, Depression, Parkinson's disease.	Nuclear receptor coactivator 2, Gamma-aminobutyric-acid receptor subunit alpha-6, Sodium-dependent noradrenaline transporter, Alpha-1A adrenergic receptor, Sodium-dependent dopamine transporter, Interleukin-6, Prostaglandin G/H synthase 1, Muscarinic acetylcholine receptor M3, Prostaglandin G/H synthase 2, Gamma-aminobutyric-acid receptor alpha-2 subunit, Retinoic acid receptor RXR-alpha, Alpha-1B adrenergic receptor, Gamma-aminobutyric acid receptor subunit alpha-1.
Limonene	Abdominal aortic aneurysm, Adenomatous polyposis, Alzheimer's Disease, Analgesics, Arthritis, Bladder cancer, Breast cancer, Cancer, unspecified , Carpal tunnel syndrome, Colorectal cancer, Dysmenorrhea, unspecified Endometriosis, Genitourinary tumors, Gestational hypertension, Inflammation, Inflammatory diseases, Lung Cancer, Malignant mesothelioma, Meningioma, Myocardial Infarction, Oropharyngeal squamous cell carcinoma, Osteoarthritis, Pain, unspecified, Pathological angiogenesis, Peutz-Jeghers syndrome, Prostate cancer, Pyresis, Renal Cell Carcinoma, Rheumatoid arthritis, unspecified, Anxiety Disorders, Insomnia, Alcoholism, Autoimmune cardiomyopathy, Chronic obstructive pulmonary disease, Neurogenic bladder, Tremor, Anxiety disorder, Cognitive deficits, Schizophrenia, Central nervous system diseases, Delirium, Dementia.	Prostaglandin G/H synthase 2, Gamma-aminobutyric acid receptor subunit alpha-1, Alcohol dehydrogenase 1B, Alcohol dehydrogenase 1C, Cytochrome P450-cam, Nuclear receptor coactivator 2, Nuclear receptor coactivator 1, Gamma-aminobutyric-acid receptor alpha-2 subunit, Muscarinic acetylcholine receptor M1, Gamma-aminobutyric-acid receptor alpha-5 subunit, Gamma-aminobutyric-acid receptor alpha-3 subunit, Ig gamma-1 chain C region, Gamma-aminobutyric-acid receptor subunit alpha-6,
